# Magnetic Resonance Imaging‐Guided Delivery of Neural Stem Cells into the Basal Ganglia of Nonhuman Primates Reveals a Pulsatile Mode of Cell Dispersion

**DOI:** 10.5966/sctm.2016-0269

**Published:** 2016-09-22

**Authors:** Kristen E. Malloy, Jinqi Li, Gourav R. Choudhury, April Torres, Shruti Gupta, Chris Kantorak, Tim Goble, Peter T. Fox, Geoffrey D. Clarke, Marcel M. Daadi

**Affiliations:** ^1^ Southwest National Primate Research Center, Texas Biomedical Research Institute, San Antonio, Texas, USA; ^2^ Research Imaging Institute, Radiology, University of Texas Health Science Center, San Antonio, Texas, USA; ^3^ MRI Interventions, Inc., Irvine, California, USA

**Keywords:** Interventional magnetic resonance imaging, Stem cell delivery, Cell flow, Nonhuman primate, ClearPoint system, Rheology, Real‐time interventional magnetic resonance imaging‐guided cell transplantation

## Abstract

Optimal stem cell delivery procedures are critical to the success of the cell therapy approach. Variables such as flow rate, suspension solution, needle diameter, cell density, and tissue mechanics affect tissue penetration, backflow along the needle, and the dispersion and survival of injected cells during delivery. Most cell transplantation centers engaged in human clinical trials use custom‐designed cannula needles, syringes, or catheters, sometimes precluding the use of magnetic resonance imaging (MRI)‐guided delivery to target tissue. As a result, stem cell therapies may be hampered because more than 80% of grafted cells do not survive the delivery—for example, to the heart, liver/pancreas, and brain—which translates to poor patient outcomes. We developed a minimally invasive interventional MRI (iMRI) approach for intraoperatively imaging neural stem cell (NSC) delivery procedures. We used NSCs prelabeled with a contrast agent and real‐time magnetic resonance imaging to guide the injection cannula to the target and to track the delivery of the cells into the putamen of baboons. We provide evidence that cell injection into the brain parenchyma follows a novel pulsatile mode of cellular discharge from the delivery catheter despite a constant infusion flow rate. The rate of cell infusion significantly affects the dispersion and viability of grafted cells. We report on our investigational use of a frameless navigation system for image‐guided NSC transplantation using a straight cannula. Through submillimeter accuracy and real‐time imaging, iMRI approaches may improve the safety and efficacy of neural cell transplantation therapies. Stem Cells Translational Medicine *2017;6:877–885*

**Significance Statement**

Optimal stem cell delivery procedures are critical to the success of the cell therapy approach. A minimally invasive interventional magnetic resonance imaging approach was developed for imaging intraoperatively neural stem cell (NSC) delivery procedures. NSC delivery was imaged in real time in nonhuman primate forebrain. Evidence indicates that cell injection follows novel pulsatile mode of dispersion and that the rate of cell infusion significantly affects the dispersion and viability of grafted cells. This approach will improve the safety and efficacy of neural transplantation and address some of the current bottlenecks in translating stem cell therapies to the clinic.

## Introduction

Optimizing stem cell delivery procedures will be critical to the success of cell therapy approaches. Unlike small molecules and growth or neurotrophic factors, stem cells are more sensitive to their microenvironment. Variables such as flow rate, needle diameter, cell density, tissue porosity, and mechanics affect the penetration and dispersion of the cells in the target tissue, and ultimately their survival and function. Most translational research and cell transplantation centers engaged in human clinical trials use standard microsyringes or long cannulas or catheters that sometimes preclude use of magnetic resonance imaging (MRI)‐guided delivery to target tissue. As a result, the full therapeutic potential of stem cell therapies may be compromised by the lack of target precision and cell death caused by suboptimal injection parameters. In fact, the majority of cells grafted (more than 80%) do not survive the delivery in the heart [1], liver/pancreas [2], and brain [3], which translates to poor results in patients. There is a critical need for a regulated preapproved device along with a standardized delivery procedure with optimal parameters to ensure cell survival, consistency, and comparability of data from multicenter clinical studies. Such approach is paramount to the success of translational stem cell therapies.

Extrapolating from work in our and other laboratories on drug and viral vector delivery into the central nervous system (CNS) [4, 5, 6, 7, 8], it has been shown that the exertion of increasing positive pressure, which creates a gradient, improves the volume of distribution (Vd) to the volume of infusion (Vi) (Vd/Vi) ratio and prevents backflow of the infusate. This approach favors dispersion of very small particles by convection rather by diffusion [9]. In addition, smaller diameter cannulas (i.e., <32 gauge) and slow injection rates (i.e., 0.5 μl per minute) are optimal parameters to prevent backflow and enhance tissue distribution for the injection of very small particles, such as drugs and viral vectors [10, 11]. The backflow is also reduced by the use of a stepped cannula design in which the cannula decreases in diameter in discrete steps approaching the tip [12, 13, 14]. These steps provide a physical barrier that reduces backflow of drugs and viral vector particles during injections. However, it’s not clear whether this approach would apply to neural cell injections into the CNS. A recent report has demonstrated MRI‐guided intracerebral transplantation of neural cells using radially branched cannula that enable multiple deposits through the same cannula insertion [15, 16].

Although injection parameters have been well‐studied and controlled for small particles, like drugs and viral vectors, the technology for stem cell delivery remains undeveloped and limited by inconsistent cell survival, and suboptimal injection accuracy and tissue penetrance. Parameters that have been optimized for drugs and viral vectors, may not apply directly to stem cell delivery although they provide a starting point.

We developed a minimally invasive interventional MRI (iMRI) approach for imaging intraoperatively a neural stem cell (NSC) delivery procedure and for achieving accurate cannula tip placement. We used NSCs prelabeled with a contrast agent and real‐time magnetic resonance imaging to guide the injection cannula to the target and to track the delivery of the cells into the putamen of baboons. We provide evidence that cell injection into the brain parenchyma follows a novel pulsatile mode of dispersion and that the rate of cell infusion significantly impacts the viability of grafted cells.

## Materials and Methods

### Neural Stem Cell Derivation and Preparation

**Figure 1:**
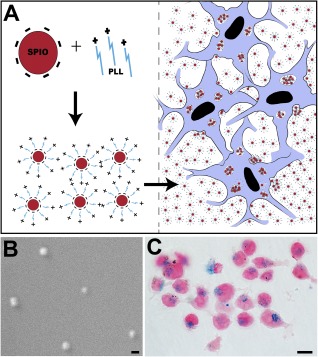
SPIO labeling of neural stem cells (NSCs). **(A):** Schematic drawing of SPIO particles’ negatively charged complexes through electrostatic interactions with the positively charged PLL molecule and take‐up (encapsulation) by the cells after incubation, thus forming intracellular endosomes. **(B):** Electron microscope scanning image showing the actual size of the SPIO nanoparticles. **(C):** Photomicrograph of NSCs after 72‐hour incubation with SPIO‐PLL complex shows intracellular SPIO nanoparticles revealed with Prussian blue staining. Scale bars = 200 nm **(B)**, 15 µm **(C)**. Abbreviations: PLL, poly‐l‐lysine; SPIO, superparamagnetic iron oxide.

Human induced pluripotent stem cells (iPSCs) were generated from human skin fibroblast procured from the NIH (ND29802; National Institute for Neurological Disorders and Stroke** **Repository, Coriell Institute, Camden, NJ, https://catalog.coriell.org/1/NINDS). We used the episomal approach previously described in detail [17]. NSCs were derived from iPSCs, as we previously described [18]. To label NSCs prior to the cell transplantation, we incubated them in a neural stem cell medium supplemented with superparamagnetic iron oxide nanoparticles (Feridex) and poly‐l‐lysine for 48 hours, as we previously described [19] (Fig. 1). The cells were collected the day of the injection, suspended in neural stem cell media at a concentration of 70,000 cells per microliter, and stored on ice until injected.

### NSC Injection Into Brain Phantom Gel

**Figure 2:**
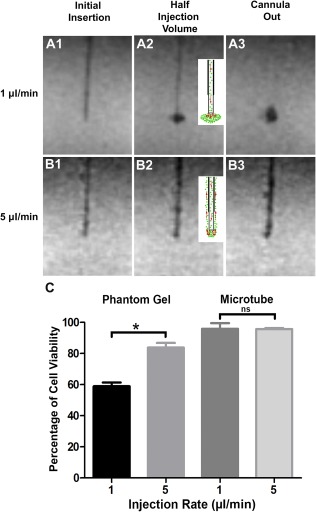
Impact of infusion rate and physical constraints on cell dispersion and survival in a brain phantom gel. Magnetic resonance imaging scans showing the progression of superparamagnetic iron oxide‐labeled neural stem cells (NSCs) infused into agarose gel phantoms at 1 μl per minute **(A1–A3)** and 5 μl per minute **(B1–B3)**. The schematic drawings **(A2, B2)** depict the flow and dispersion (red arrows) of cells (1 μl per minute) **(A2)** and backflow of cells up the cannula trajectory (5 μl per minute) **(B2)**. The postinjection scans (cannula out) show a well‐formed cloud of NSCs at 1 μl per minute **(A3)** and backflow of NSCs along the cannula trajectory at 5 μl per minute **(B3)**. **(C):** NSCs injected either into the phantom gel or into the air inside a microtube show that slow injection (1 µl per minute) causes more cell death than does faster injection rate (5 µl per minute), although no significant change in viability was observed when NSCs were injected into a microtube. Data represent the mean ± SEM of experiments performed in triplicate in three independent experiments. ∗, *p* < .05. Abbreviation: ns, not significant.

The superparamagnetic iron oxide (SPIO)‐labeled NSCs were infused into 0.6% agarose gel (Fig. 2) using an MRI‐compatible cannula with an inner diameter of 530 μm (NeoNeuron LLC, Palo Alto, CA, http://neoneuron.org/index.html) connected to a 25‐ to 100‐μl microsyringe (SGE Analytical Science, Victoria, Australia, http://www.sge.com) via a polyurethane infusion line (VAHBPU; Instech Laboratories, Plymouth Meeting, PA, http://www.instechlabs.com). These experiments were performed in triplicate in three independent experiments. The microsyringe was positioned in an MRI‐compatible syringe pump (Chemyx, Stafford, TX, https://www.chemyx.com). The volume cell suspension needed for injection was loaded at the tip of the cannula using the MRI‐compatible microsyringe pump. Once the volume of cell suspension needed was uploaded, the cannula was immediately inserted into the brain, and cell infusion started. The cell infusion was monitored using a repeating single‐slice TurboFLASH sequence (echo time [TE] = 2.25 millisecond, repetition time [TR] = 1,700 millisecond, 0.523 × 0.523 × 2.5 mm, 128 × 128 × 1 pixels, number of excitations [NEX] = 1, flip angle = 5°). The image series was initiated prior to cannula insertion and continued for the duration of each injection for the two injection rates tested: 20‐μl volume at 1 μl per minute and 20‐μl volume at 5 μl per minute. The cannula was removed 5 minutes after the completion of cell injection.

Following injection into the phantom the viability of the cells was determined by dye exclusion method using 0.4% trypan blue solution (Thermo Fisher Scientific Life Sciences, Waltham, MA, http://www.thermofisher.com). The cells were collected from the phantom and suspended in fresh media. A sample of the cell suspension was mixed with an equal volume of trypan blue and loaded on to a hemocytometer. Viable cells with clear cytoplasm were counted and expressed as a percentage of total cells (live and dead cells). GraphPad prism software was used to plot the graphs and perform statistical analysis.

### MRI‐Guided Injection of NSCs Into the Baboon Basal Ganglia

We used the ClearPoint system (MRI Interventions, Irvine, CA, http://www.mriinterventions.com) for the iMRI‐guided delivery of NSCs. ClearPoint is a frameless navigation system for intraoperative MRI that uses fiducial placed on a head‐mounted aiming device.

#### Determination of the Entry Point

**Figure 3:**
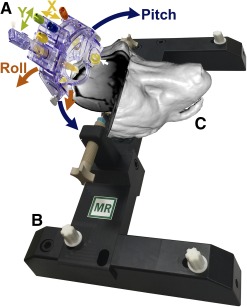
ClearPoint system used for interventional magnetic resonance imaging (iMRI)‐guided transplantation of neural stem cells. **(A):** The ClearPoint is a frameless navigation system that uses fiducials (white arrow) placed on a head‐mounted aiming device. The system includes the SmartFrame trajectory guide **(A)**, which is hand controlled to rotate and align the cannula guide using initially the blue (pitch; blue arrows) and orange (roll; orange arrows) knobs, followed by the yellow (X; yellow arrows) and green (Y; green arrows) knobs for the final fine adjustments. **(B):** Head‐fixation frame. **(C):** Three‐dimensional (3D) surface rendering of a representative baboon head positioned in the head‐fixation frame locked to the MRI table. The surface rendering was constructed from a postinjection MRI series using the “build surface” feature in the software Mango. The 3D surface was smoothed and overlaid with the original MRI slices at the cut planes corresponding to the injection site. Abbreviation: MR, magnetic resonance.

An MRI‐compatible head‐fixation frame (Fig. 3) was fastened into the notches on the sides of the MRI table and placed close to the bore of the magnet, a Siemens Trio 3T system. The cadaveric baboon heads (*n* = 3) were secured into the head‐fixation frame, oriented in a supine position. The SmartGrid is a 6 × 6 array of MRI‐sensitive gadolinium‐filled squares used in the treatment‐planning software to determine the optimal cannula entry point to the target structure. It was placed on the skull, covering the estimated entry point of the needle. A T1‐weighted three‐dimensional (3D) gradient echo image (TE = 2.6 millisecond, TR = 14 millisecond, 1 × 1 × 1 mm, 320 × 320 × 208 pixels, NEX = 1, flip angle = 15°) was acquired and transferred to the ClearPoint software via Digital Imaging and Communications in Medicine network. The software automatically detected the anterior commissure (AC), posterior commissure (PC), and midsagittal plane to position the brain in a 3D coordinate system. The injection target was selected and cannula trajectory was established, and the software calculated the intersection of the cannula trajectory with the SmartGrid. The target access was checked to confirm that the injection site is reachable from the selected entry point, and corresponding coordinates of the SmartGrid for this entry point were used to drill a burr hole.

#### SmartFrame Alignment

The base of the SmartFrame was positioned directly over the burr hole and secured to the skull with three self‐taping screws fixed to the base. The SmartFrame tower is then mounted to the base. A hand controller with matching colored knobs was mounted on the SmartFrame to adjust the cannula from a distance without moving the head from MRI isocenter. The baboon head was then moved to the MRI isocenter. A 3D T1‐weighted gradient echo series was repeated and transferred to the ClearPoint workstation, ensuring that the four MRI‐sensitive fiducial markers of the SmartFrame were included in the series. (TE = 2.6 millisecond, TR = 14 millisecond, 1 × 1 × 1 mm, 320 × 320 × 208 pixels, NEX = 1, flip angle = 15°). The software segmented the SmartFrame fiducial markers and used their locations to calculate the scan plane parameters necessary to acquire a single‐slice T1‐weighted two‐dimensional (2D) image aligned axially with the planned trajectory of the cannula and in the plane of the cannula guide tip (TE = 6.8 millisecond, TR = 500 millisecond, 3.906 × 3.906 × 5 mm, 128 × 128 × 1 pixels, NEX = 1, flip angle = 180°). The ClearPoint software used the resulting image slice to calculate pitch and roll SmartFrame adjustments to correct cannula angle and match the planned trajectory. The SmartFrame hand controller was used to rotate the pitch (blue) and roll (orange) thumb wheels. The same single‐ slice 2D image was acquired, and this procedure was repeated until the cannula alignment error was within an acceptable range.

#### Finalizing Trajectory

The software calculated the scan plane parameters needed to acquire two orthogonal 3D image series aligned axially with the trajectory of the cannula (TE = 9.5 millisecond, TR = 1,530 millisecond, 1 × 1 × 1 mm, 256 × 256 × 15 pixels, NEX = 2, flip angle = 152°). For the first “Align adjustment” set, software calculated the required adjustments for pitch and roll directions. The second scan, the “Off‐set adjustment,” used the X and Y (yellow and green) knobs, respectively. For both Align and Offset adjustments the corrections were made, and additional scans were reacquired and corrections made until the cannula alignment error was within an acceptable range (<1 mm^3^). Once the trajectory was finalized, the software calculated the cannula depth to reach the target. A depth stopper was attached to the cannula at this position.

#### NSC‐SPIO Injection Into the Putamen

The pump was used to withdraw 20 μl of NSC suspension. The infusion line was primed, and the injection began at 1 μl per minute. The cannula was slowly lowered into the brain tissue until it reached the depth stop. The injection was initiated and monitored using a repeated single‐slice TurboFLASH image series (TE = 2.25 millisecond, TR = 1,700 millisecond, 0.523 × 0.523 × 2.5 mm, 128 × 128 × 1 pixels, NEX = 1, flip angle = 5°). The TurboFLASH series was initiated during the injection and ended 5 minutes after end of the injection upon the removal of the cannula. Two postinjection sequences were acquired to visualize the graft, a 3D T1‐weighted gradient echo series (TE = 2.6 millisecond, TR = 14 millisecond, 1 × 1 × 1 mm, 320 × 320 × 208 pixels, NEX = 1, flip angle = 15°) and a coronal Turbo Spin Echo series (TE = 83 millisecond, TR = 3,000 millisecond, 0.938 × 0.938 × 1.2 mm, 128 × 128 × 27 pixels, flip angle = 131°)

### Histopathology, Immunocytochemistry, and Microscopical Analysis

The cadaveric baboon brains were extracted from the skull; postfixed in 4% paraformaldehyde; cryoprotected in an increasing gradient of 10%, 20%, and 30% sucrose solution; and cryostat‐sectioned at 40 µm. For Prussian blue staining, sections were fixed using 2% glutaraldehyde, washed in phosphate‐buffered solution (PBS), and incubated with Pearls reagent (4% potassium ferrocyanide per 12% HCL, 50:50 volume) for 40 minutes under agitation. Sections were then washed in PBS and twice in water, dehydrated through graded alcohols, and counterstained with nuclear fast red. For immunocytochemistry, brain sections were rinsed in PBS for 3 × 5 minutes, then incubated overnight with the STEM121 (1:200; StemCells Inc., Newark, CA, http://www.stemcellsinc.com) primary antibody. Secondary antibodies raised in mouse conjugated to fluorescein isothiocyanate, rhodamine‐B‐isothiocyanate, aminomethylcoumarin acetate, CY3, or CY5 chromogenes (Jackson ImmunoResearch, West Grove, PA, https://www.jacksonimmuno.com) were used. Sections were counterstained with the nuclear marker 4´,6‐diamidino‐2´‐phenylindole dihydrochloride. Positive and negative controls were included in each run. Immunostained sections were coverslipped using fluorsave (Calbiochem) as the mounting medium and analyzed using the LSM‐800 ZEISS laser scanning confocal microscope.

#### Terminal Deoxynucleotidyl Transferase 2´‐Deoxyuridine, 5´‐Triphosphate Nick‐End Labeling Assay

Terminal deoxynucleotidyl transferase (TdT) 2´‐deoxyuridine, 5´‐triphosphate nick‐end labeling (TUNEL) was done using the TACS 2 TdT‐3,3′‐diaminobenzidine (DAB) in situ apoptosis detection kit (R&D Systems, Minneapolis, MN, https://www.rndsystems.com) to quantify the apoptosis in the graft. Briefly, the frozen sections were hydrated and treated with proteinase K solution before incubating with TdT end‐ labeling cocktail. The sections were then rinsed and incubated with streptavidin‐horseradish peroxidase and treated with DAB solution. We used 1% methyl green to counterstain nuclei of viable cells. Images were taken with Nikon eclipse te2000 and analyzed using Image‐J software. The number of TUNEL+ cells was determined by counting the number of cells at ×63 magnification in 15 randomly chosen microscopic observation fields per graft section. Within the same randomly chosen fields and at the same magnification, the total numbers of live cell nuclei stained with methyl green were also determined. The total counts were then expressed as percentage of the total methyl green stained nuclei.

### Statistical Analysis

Outcome measurements for each experiment were reported as mean ± SEM. The data were analyzed using GraphPad prism software. Significance of intergroup differences was performed by applying Student’s *t* test where appropriate. One‐way analysis of variance was used to compare group differences. Differences between the means were determined using Bonferroni’s post hoc test. A *p* value of less than .05 was considered to be statistically significant.

## Results

### Real‐Time MRI Monitoring of NSC Injection Into a Simulated Brain Phantom Gel

To investigate the effects of injection rate and the microenvironment on cell survival and dispersion, we used a surrogate brain model consisting of 0.6% agarose gel [20, 21, 22, 23, 24] with real‐ time MRI. The gel formulation offers poroelastic material with mechanical properties and hydraulic conductivity similar to that of soft brain tissue and has been previously used to test various drug delivery parameters and to understand the biophysics of tissue penetration and backflow of infusate [23, 24, 25, 26]. To visualize the cell injection in real time under MRI, we labeled the NSCs with superparamagnetic iron oxide (SPIO), a clinical‐grade magnetic resonance contrast agent. SPIO nanoparticles (Fig. 1) were first mixed and incubated with poly‐l‐lysine (PLL) for an hour, and the SPIO‐PLL mixture was then used to treat cultured NSCs for 3 days in vitro (Fig. 1). The NSCs were infused at two different rates, 1 μl per minute and 5 μl per minute and magnetic resonance imaged using TurboFLASH sequence (Materials and Methods section).

The injection at 1 μl per minute resulted in cell dispersion and formation of a cloud at the tip of the cannula with no evidence of cell backflow along the cannula track (Fig. 2A). The cells were observed to flow in a pulsatile manner with multiple (four) distinct bursts of cells, three occurring intermittently throughout the injection and one upon removal of the cannula (supplemental online Video 1). This was consistent in multiple trials. Interestingly, in every trial the cells appeared to flow laterally from the cannula into the phantom gel and initially formed an oval‐shaped cloud (Fig. 2A2; supplemental online Video 1).

The infusion at 5 μl per minute showed a similar pulsatile flow but with fewer (two) bursts of cells, one during infusion and one upon removal of the cannula (supplemental online Video 2; Fig. 2B). However, at this rate, the cells did not penetrate and disperse into the phantom gel nor did they form a cloud at the tip; instead, there was a backflow of NSCs during the injection along the cannula track.

To determine the impact of the infusion pressure and shear forces on cell survival, we collected the infused NSCs and determined their viability in triplicate in three independent experiments. Interestingly, the cell viability was significantly reduced at the slow 1 μl per minute rate versus the 5 μl per minute rate in the phantom gel in comparison with injection in an Eppendorf microtube (Fig. 2C). The injection of the NSCs into the Eppendorf microtube demonstrated that neither the physical infusion (pass‐through cannula) nor the rate of infusion (1 versus 5 μl per minute) affected the viability of the cells (Fig. 2C).

### MRI‐Guided Delivery of NSC‐SPIO Into the Putamen of Baboon

**Figure 4:**
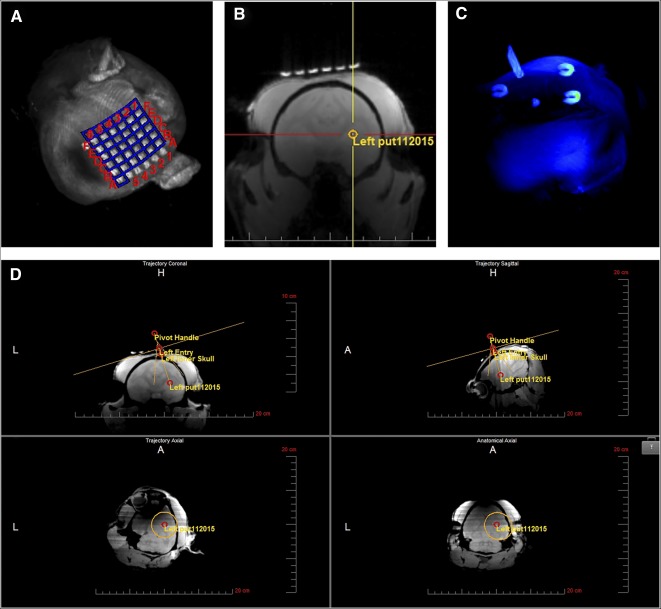
Trajectory alignment. **(A):** Maximum intensity projection (MIP) of the SmartGrid, a 6 × 6 array of magnetic resonance imaging (MRI)‐sensitive gadolinium‐filled squares placed on the skull covering the estimated entry point of the needle. **(B):** Three‐dimensional T1‐weighted gradient echo series transferred to the ClearPoint workstation and used to select the target. **(C):** MIP of the SmartFrame trajectory guide showing fiducial markers used by the software to segment the SmartFrame and to calculate magnetic resonance scanning parameters for planning the trajectory of the cannula. **(D):** Point of entry and trajectory to the target set based on the anterior and posterior commissures and the midsagittal plane and adjusted, if needed, to ovoid particular structures, ventricular systems, or blood vessels using the “fly‐through” trajectory option. Abbreviations: A, anterior; H, head; L, left.

**Figure 5:**
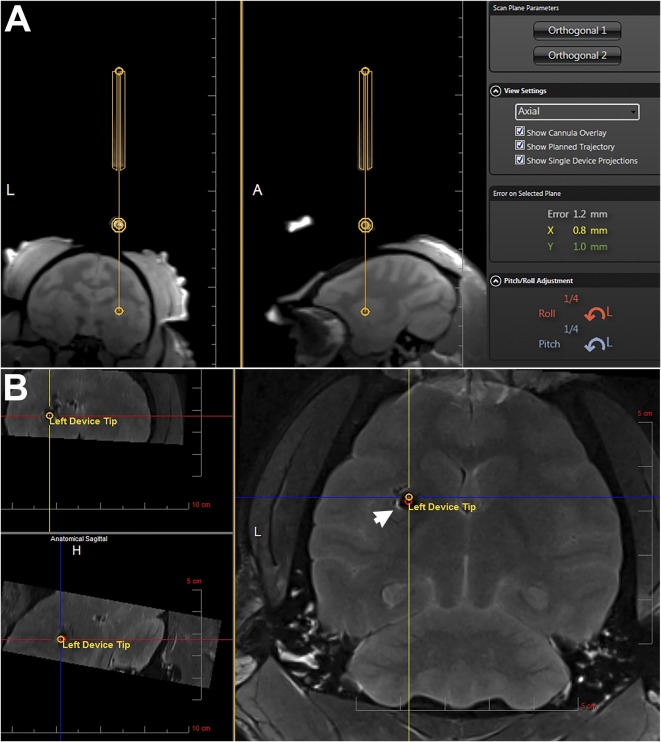
Finalizing trajectory and cannula entry. **(A):** Representative slices of the orthogonal alignment images shown in the ClearPoint software. The yellow lines outline the segmentation of the cannula guide. The software‐calculated adjustments are shown in orange (Roll) and blue (Pitch) on the bottom right. **(B):** Postinjection magnetic resonance image overlaid with the planned target and cannula tip (Left Device Tip) as calculated by the ClearPoint software showing the superparamagnetic iron oxide‐labeled neural stem cell graft (arrow, hypointense area) on target. Abbreviations: A, anterior; H, head; L, left.

The cadaveric baboon heads (*n* = 3) were positioned in a supine position in an magnetic resonance‐compatible fixation frame (Fig. 3) for the identification of the entry point of the cannula to the putamen and the skull area to mount the frameless device (Fig. 3). A maximum‐intensity projection of the 3D T1‐weighted series acquired with the SmartGrid (Fig. 4A) was used to identify the entry point during the planning phase. Once the target was selected (Fig. 4B), the software determined the trajectory (Fig. 4C, 4D) to pass through rows A–F, columns 1–6, of the SmartGrid (Fig. 4A). To align the SmartFrame with the skull, we performed a maximum intensity projection image (Fig. 4C) rendered from the MRI scans showing the four fiducial markers. Three of the fiducial markers distributed in an axial plane were detected by the software and used to calculate the SmartFrame adjustments. The software used the forth fiducial marker at the tip of the cannula guide to determine the cannula trajectory. We took multiple 2D T1 alignment images to align the cannula guide trajectory with the planned trajectory using the pitch and roll adjustments (Fig. 3). Two orthogonal 3D images (Fig. 5A) enabled the software to segment the cannula guide, and final adjustments to the pitch, roll, and X and Y offsets were made (Fig. 5).

Once the error of cannula trajectory was less than 1 mm, the NSCs labeled with SPIO were uploaded to the MRI‐compatible needle and positioned for entry into the basal ganglia of the baboon brain. The software calculated the depth to the target structure, and a depth stop was fixed to the cannula at the determined depth.

To image and monitor the NSC injections in real time, we used the TurboFLASH time series (Materials and Methods section). We selected 1 µl/ml because we found that this injection rate promoted cell dispersion and eliminated backflow of cells up along the cannula track. A 20‐μl cell suspension was injected over 20 minutes. The results showed similar pulsatile mode of NSC dispersion into the brain parenchyma, as we observed using the brain phantom. The real‐time MRI showed little evidence of cell suspension flowing in the beginning of the infusion followed by a pulsatile injection of cells appearing at the tip of the cannula after approximately 10 minutes of the infusion time (supplemental online Video 3).

**Figure 6:**
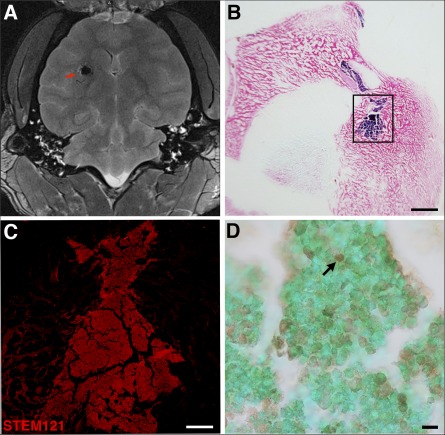
Postinjection magnetic resonance image and immunohistochemistry of superparamagnetic iron oxide (SPIO)‐labeled neural stem cells (NSCs). **(A):** MRI shows the SPIO‐labeled NSCs as a hypointense region indicated by the red arrow on the horizontal T2‐weighted scan. **(B):** Image of Prussian blue staining of a frontal section of the brain counterstained with nuclear fast red, visualizing the NSC graft in blue. **(C):** Inset from **(B)** showing high‐power immunofluorescence staining of the NSC graft with the human marker STEM121. **(D):** Terminal deoxynucleotidyl transferase 2´‐deoxyuridine, 5´‐triphosphate nick‐end labeling assay staining of NSC grafts in baboon brain showing dead cells (black arrow) inside the graft and live cell nuclei stained with methyl green. Scale bars =2.5 mm **(B)**, 500 µm **(C)**, and 20 µm **(D)**.

The grafted SPIO‐labeled NSCs were readily visible in a T2‐weighted magnetic resonance image as a hypointense area (Figs. 5B, 6A). The accuracy of the injection is evident in Figure 5B with the overlay of the planned trajectory (red circle) and device tip (yellow circle) locations clearly within the cell graft.

### Immunohistopathological Confirmation of the NSCs’ Graft Identity and Target Location

To confirm the location of the NSC graft, we sectioned brains and processed them for histology and immunocytochemistry. SPIO‐labeled NSC grafts were readily detected with Prussian blue staining in the putamen target structure (Fig. 6B), confirming the MRI data. Immunostaining with human marker STEM121 and confocal analysis confirmed the human NSC graft identity and location (Fig. 6C). To determine viability inside the grafts, we processed brain sections for TUNEL assay and counterstained them with the live cell marker methyl green. Using the 1 µl per minute infusion rate, the analysis revealed that 25.1% ± 3.2% of grafted cells died after grafting (Fig. 6D).

## Discussion

Stem cell‐based therapy is emerging as a promising treatment for a variety of diseases and injuries. The first step in evaluating the potential of different therapeutic stem cell lines is to develop a safe and effectively reproducible delivery system. Real‐time iMRI‐guided delivery of cellular products into the target tissue is critical to the success of the therapy. A noninvasive iMRI approach is becoming a necessity in the clinical application to enhance the safety of patients and the efficacy of the therapeutic approach. Currently, the effects of flow rate, infusion pressure, and shear forces imposed by the physical properties of the microenvironment on cell survival, penetration, and dispersion are not well documented. These translational bottlenecks are significant because even with the best cell preparation, failure of the product to show efficacy may be simply due to a compromised delivery approach. Thus, interventional delivery parameters can in fact make or break a clinical study.

We report optimal parameters affecting cell dispersion and survival in brain phantom gel and the application of these parameters in nonhuman primate target brain structure using minimally invasive iMRI‐guided NSC transplantation into the brain. This procedure was realized using the ClearPoint system with frameless SmartFrame stereotaxic fiducial‐based navigation that achieved accurate cannula tip placement of iPSC‐derived NSC grafts. We describe a novel pulsatile mode of cell dispersion into both the brain phantom gel and brain parenchyma. NSCs prelabeled with SPIO were magnetic resonance‐imaged in real time during the iMRI‐guided injection process to the putamen of baboon and location was confirmed by immunohistopathological approach. We found that the rate of injection affected both the dispersion and survival of the NSCs in the brain phantom gel. The injection rate of 1 μl per minute led to a comparable viability of approximately 60% and 75% in the phantom gel and baboon brain, respectively. The difference may be attributed to the physiologically supportive and neurotrophic environment in the brain parenchyma in comparison with the synthetic phantom gel.

Our results suggest that cell infusion and dispersion in brain phantom gel and brain parenchyma follow a pulsatile mode, implying that unlike drugs, cells do not flow smoothly at a specified rate but rather are emitted in small bursts. This may be caused by the buildup and release of pressure caused by the cells progressing through the infusion line. Indeed, we found that using injection cannula of 200‐ to 300‐µm diameter or lower with a 10‐foot‐long infusion line caused an increased pressure with no cell delivery during the intended period (data not shown). The cells remained blocked in the cannula during the total duration of infusion (20 minutes). It is plausible that the density of cell suspension medium may play an important role in rheology and cell buoyancy, which may minimize cell sedimentation and facilitate the flow of cells. In this study, we used cell culture medium in which cells can sediment if left for an extended period of time. To minimize the sedimentation, we loaded NSCs to the injection needle just before insertion in the brain. In addition, the supine position of the baboon head minimized the effects of gravity on cell sedimentation in the needle. More viscous cell suspension solutions may reduce shear forces, adherence, clumping, and sedimentation and improve rheology and cell survival [27, 28].

Rate of infusion plays an important role in the distribution of therapeutics in the target tissue. Convection‐enhanced delivery has been shown to be an effective method for the direct injection of therapeutic agents into the brain [9, 10]. This method uses positive pressure to deliver an infusate by convection as opposed to diffusion, which because of the pressure gradient helps to both increase the Vd/Vi ratio and prevent backflow. It has been shown that smaller‐diameter cannulas (<32 gauge) and slow injection rates (<1 μl per minute) are ideal parameters for injection [10]. Chen et al. reported that backflow of C‐albumin in the rat model was significantly reduced with injection rates slower than 1 μl per minute and with a 28‐gauge or smaller cannula [11]. Backflow is also reduced by the use of a stepped cannula design, in which the cannula decreases in diameter in discrete steps approaching the tip [12, 13]. These steps provide a physical barrier that reduces backflow. Other types of cannulas were also designed with the idea of decreasing backflow of cells, such as radially branched cannula, that enable the use of the ClearPoint system with access to multiple sites through the same insertion track [16]. Although the parameters established in gene therapy and drug‐based experiments provide a good starting point for stem cell therapy, the ideal parameters for stem cell therapy have yet to be defined.

## Conclusion

The ClearPoint iMRI‐based navigation platform initially developed for iMRI‐guided deep brain stimulation (DBS) [29, 30, 31, 32] has received clearance in the United States and the European Union for clinical use and has significantly improved clinical outcome for DBS patients [31, 32]. The system has also been used for (a) drug infusion into gliomas and for adeno‐associated viral vector‐expressing glial‐derived neurotrophic factor into Parkinsonian patients [33], (b) brain biopsies [34, 35], (c) targeted ablation in epilepsy [36], and (d) the BranchPoint Radially Branched Deployment device for stem cell delivery to large brain targets, which is in development for human use [16]. We report the use of the ClearPoint system with an MRI‐compatible straight injection cannula that eliminates backflow for iMRI‐guided NSC transplantation. With the ClearPoint system, this cannula allowed for monitoring and avoiding unwanted structures, such as blood vessels, and for preventing hemorrhages during cell transplantation. This iMRI approach may improve the safety and efficacy of neural cell transplantation therapy.

## Author Contributions

K.E.M.: collection and/or assembly of data, data analysis and interpretation, manuscript writing; J.L., G.R.C., A.T., S.G., C.K., and T.G.: collection and/or assembly of data; P.T.F. and G.D.C.: administrative support, provision of study material; M.M.D.: conception and design, financial support, administrative support, provision of study material, collection and/or assembly of data, data analysis and interpretation, manuscript writing, final approval of manuscript.

## Disclosure of Potential Conflicts of Interest

S.G., C.K., and T.G. are employees of MRI Interventions, Inc. M.M.D. is founder of NeoNeuron, a biotechnology company. The other authors indicated no potential conflicts of interest.

## Supporting information

Supporting Information

Supporting Information

Supporting Information

Supporting Information
