# Supplementary material for: Magnetic Resonance Imaging‐Guided Delivery of Neural Stem Cells into the Basal Ganglia of Nonhuman Primates Reveals a Pulsatile Mode of Cell Dispersion
Source: Stem Cells Transl Med. 2016 Sep 22;6(3):877–85. doi: 10.5966/sctm.2016-0269 (PMC5442780; doi:10.5966/sctm.2016-0269)
Supplement: Supplementary file 1 — Supporting Information [file SCT3-6-0877-s001.pdf]

Video Legends – Daadi et al.

Video 1. Real-time MR imaging of SPIO-labeled NSC injection into agarose gel phantom at a rate of 1 μL/min

Video 2. Real-time MR imaging of SPIO-labeled NSC injection into agarose gel phantom at a rate of 5 μL/min

Video 3. Real-time MR imaging of SPIO-labeled NSC injection into brain parenchyma of baboon at a rate of 1 μL/min
